# Brainstem intraparenchymal schwannoma with genetic analysis: a case report and literature review

**DOI:** 10.1186/s12920-021-01049-z

**Published:** 2021-08-18

**Authors:** Daiichiro Ishigami, Satoru Miyawaki, Hirofumi Nakatomi, Shunsaku Takayanagi, Yu Teranishi, Kenta Ohara, Hiroki Hongo, Shogo Dofuku, Taichi Kin, Hiroyuki Abe, Jun Mitsui, Daisuke Komura, Hiroto Katoh, Shumpei Ishikawa, Nobuhito Saito

**Affiliations:** 1 Department of Neurosurgery, Faculty of Medicine, The University of Tokyo, 7-3-1 Hongo, Bunkyo-ku, Tokyo, 113-8655 Japan; 2 Department of Pathology, Graduate School of Medicine, The University of Tokyo, Tokyo, Japan; 3 Department of Molecular Neurology, Graduate School of Medicine, The University of Tokyo, Tokyo, Japan; 4 Department of Preventive Medicine, Graduate School of Medicine, The University of Tokyo, Tokyo, Japan

**Keywords:** Intraparenchymal schwannoma, Oncology, Genetics, Case report

## Abstract

**Background:**

Schwannomas are neoplasms that typically arise from the myelin sheath of peripheral nerves and rarely originate within the brain parenchyma. Some case reports present schwannomas arising from the brainstem, but regrowth of the tumor and the efficacy of postoperative irradiation have not been examined. In addition, the genetic background of schwannomas arising from the brainstem has not been investigated.

**Case presentation:**

A 21-year-old male presented with diplopia, dysphagia, and left-sided hemiparesis, dysesthesia, and ataxia. Intracranial imaging showed a heterogeneous mass with a cystic lesion in the pontomedullary junction. Since the tumor caused obstructive hydrocephalus, the patient underwent subtotal tumor resection. A histopathologic evaluation aided a diagnosis of brainstem intraparenchymal schwannoma. Gradual postoperative mass regrowth was recognized. Three-dimensional conformal radiotherapy was performed on the residual mass and surgical cavity. No tumor regrowth was observed 4 years after surgery. To investigate the genetic background of the tumor, target sequences for 36 genes, including *NF2*, *SMARCB1*, and *LZTR1*, and microsatellite analysis for loss of 22q did not show any somatic variants or 22q loss.

**Conclusions:**

We suggest that brainstem schwannomas might differ from conventional schwannomas in their genetic background.

## Background

Schwannomas arise from the myelin sheath of peripheral nerves and account for 8–10% of all intracranial tumors [1]. They are usually encountered in extra‑axial locations. Intra‑axial schwannomas are rare and arouse significant histopathological curiosity because they account for less than 1% of intracranial schwannomas. They have been reported in periventricular regions and in the cerebellum, but brainstem intraparenchymal lesions are rarely reported [2–4]. In addition, no reports have genetically examined brainstem intraparenchymal schwannomas. In this study, we report a case of pontomedullary intraparenchymal schwannoma and discuss its histological origins and genetic background.

## Case presentation

### History and examination

**Fig. 1:**
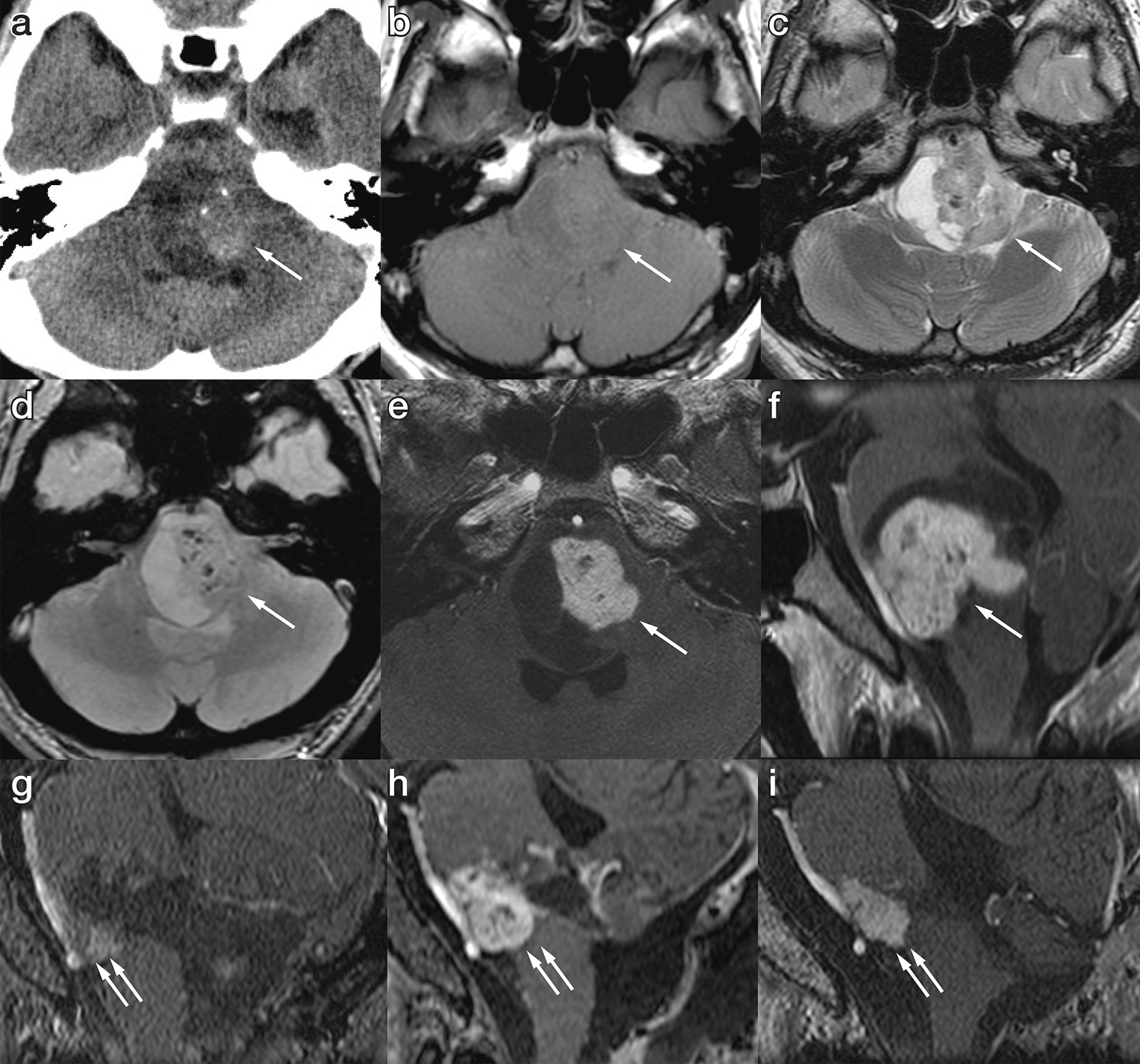
Computed tomography (CT) and magnetic resonance imaging (MRI) performed on admission. **a** Non-contrast CT, **b** T1-weighted imaging, **c** T2-weighted imaging, **d** T2* imaging, **e** gadolinium-enhanced T1 imaging (axial), and **f** sagittal reconstructed imaging. The tumor comprised a solid and a cystic component. The solid part was slightly calcified. The posterior wall of the cyst was adjacent to the floor of the fourth ventricle. **g** Gadolinium-enhanced T1-weighted imaging (Gd-T1WI) in the sagittal plane was performed on postoperative day 1, which showed the residual tumor (double arrows). **h** Gd-T1WI in the sagittal plane performed on postoperative day 43 showed marked enlargement of the gadolinium-enhanced solid component. **i** Gd-T1WI in the same slice as (**g**, **h**), which was obtained 4 years after tumor resection and irradiation. Slight shrinkage of the solid component was observed. The arrow indicates the tumor in (**a**–**f**), and the double arrows point to the postoperative residual tumor in (**g**–**h**)

A 21-year-old male without any notable past medical history presented to our hospital with numbness in the left upper extremity. Symptoms emerged a few months before the patient presented at our hospital and had been gradually deteriorating. Upon admission, a neurological examination revealed bilateral abducent palsy with partial impairment of adduction in the right eye when the patient looked to the left (one and a half syndrome), dysphagia, and left-sided hemiparesis, dysesthesia, and ataxia. His family history, including schwannomas and meningiomas, was unremarkable. Brain magnetic resonance imaging (MRI) showed a 4-cm mass in the dorsal pontomedullary area adjacent to the fourth ventricle (Fig. 1a–f). The contrast-enhanced mass was accompanied by a large cyst behind the solid component. No contrast enhancement was observed on the cyst wall. There were no other lesions such as vestibular schwannoma or spinal schwannoma in this patient. With ^18^F-fluorodeoxyglucose positron emission tomography, uptake in the lesion was non-significant, indicating minimal or no malignancy. A thorough survey of malignancy in the trunk did not reveal any lesions. Preoperative diagnostic angiography did not show tumor staining, which suggested a differential diagnosis of pilocytic astrocytoma. The fourth ventricle partially collapsed due to the mass effect, and signs of intracranial hypertension caused by obstructive hydrocephalus gradually emerged; thus, urgent tumor resection was planned.

**Fig. 2:**
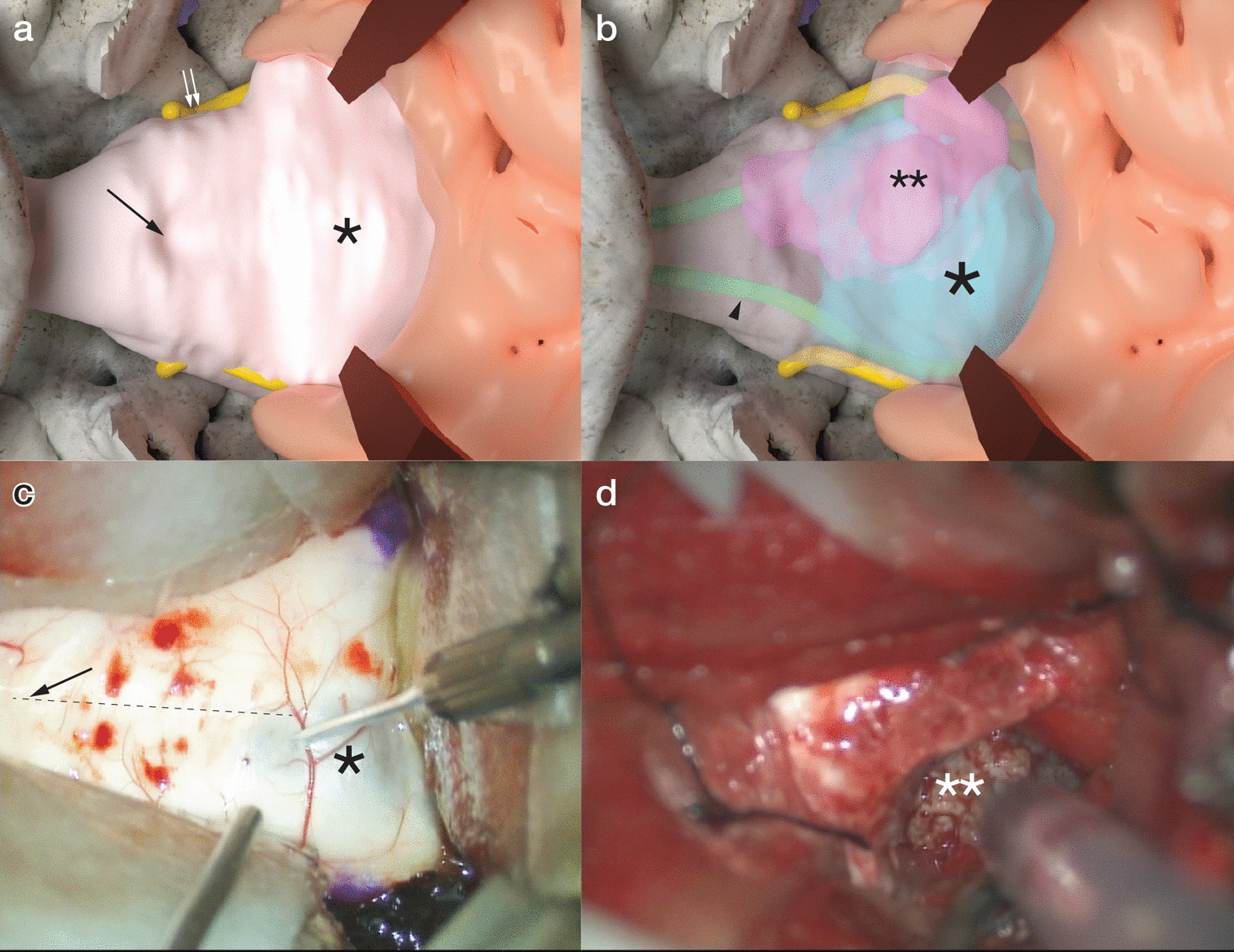
Preoperative three-dimensional evaluation of tumor components and adjacent structures. **a** The simulated operative view from the posterior side. The cerebellum is retracted to inspect the floor of the fourth ventricle. A black arrow indicates the obex, and yellow structures indicate the spinothalamic tracts (white double arrows). The asterisk is the cystic component of the tumor bulging into the floor of the fourth ventricle. **b** The simulated operative view with the translucent brainstem. The pyramidal tract is illustrated by a green line (arrowhead). The asterisk is the same as in (**a**). The double asterisks indicate the solid component of the tumor. **c** The actual operative view. The right side in this view is the rostral side of the patient. Before incision of the floor of the fourth ventricle, bilateral facial colliculi were marked by crystal violet after verification with electronic stimulation. The dashed line indicates the patient’s midline. **d** The orientation of this operative view is the same as in (**c**). The operator suctioned the solid component of the tumor (double asterisks) using an ultrasonic aspirator

The location of the motor and sensory tracts was confirmed by tractography with diffusion tensor imaging and Q-ball imaging prior to surgery. The motor and sensory tract was anteriorly and laterally compressed by the tumor, respectively. An approach from the posterior side was planned, based on the results of tractography (Fig. 2a, b).

### Surgery

A midline suboccipital craniotomy with a transcerebellomedullary fissure approach was performed. Immediately after the craniotomy, an external ventricular drain was inserted. The posterior wall of the cystic component was visible along the midline of bilateral facial colliculi (Fig. 2c). After suction of the yellowish liquid from the cyst, the anterior solid component of the tumor was identified.

Bradycardia and hypotension occurred during removal of the ventrocaudal component of the tumor, which might have been due to stimulation of the dorsal nucleus of the vagus nerve. Thus, the procedure was terminated, resulting in incomplete removal of the tumor.

### Postoperative course

A tracheotomy was performed immediately after tumor removal to prevent postoperative respiratory adverse events. Obstructive hydrocephalus resolved after tumor resection, and external ventricular drainage was removed on postoperative day (POD) 10. The ventrocaudal residual mass was followed up with gadolinium-enhanced MRI (Fig. 1g). The image on POD 29 showed slight enlargement of the contrast-enhanced area. The radiographical change was initially regarded as a postoperative inflammatory reaction, but MRI performed on POD 43 showed apparent growth of the solid component (Fig. 1h). Local fractionated irradiation was initiated at 50 Gy/25 Fr from POD 50. Subsequent follow-up MRI demonstrated no tumor regrowth. Three months after surgery, the tracheostomy was closed, and the patient was transferred to a rehabilitation facility. Left-sided facial paralysis and abducent palsy persisted even after discharge from the facility, but left-sided hemiparesis improved. Currently, no tumor regrowth has been observed 4 years after surgery (Fig. 1i).

## Methods

### Pathological and immunohistochemical investigation

A representative formalin-fixed paraffin-embedded tissue block was chosen for hematoxylin–eosin staining and immunohistochemistry, and sections with 3-µm thickness were prepared. Immunohistochemistry was performed with Ventana Benchmark (Roche, Basel, Switzerland) as the manufacturer's protocols. Primary antibodies used were as follows: anti-S100 (rabbit polyclonal, prediluted, Roche), anti-epithelial membrane antigen (EMA) (mouse monoclonal, clone E29, prediluted Roche), anti-glial fibrillary protein (GFAP) (rabbit polyclonal, Nichirei, Tokyo, Japan), Ki-67 (rabbit monoclonal, clone 30-9, Roche). S100 was evaluated in nuclei and cytoplasm. EMA was evaluated at the cell membrane. GFAP was evaluated in cytoplasm. Ki-67 labeling index was calculated as percent positive tumor nuclei.

### Target sequencing

To reveal the genetic background of the tumor, target sequences were performed on all exons in the 36 selected genes from the tumor and germline DNA, including *NF2*, *SMARCB1*, and *LZTR1*, using HaloPlexHS targeted capture (Agilent Technologies, Santa Clara, USA). Targeted genes were *NF2*, *SMARCB1*, *LZTR1*, *ALPK2*, *APC*, *ARID1A*, *ARID1B*, *BAP1*, *BRAF*, *BRCA2*, *CABIN1*, *CAST*, *CDKN2A*, *CDKN2B*, *COQ6*, *DDR1*, *EPB41L3*, *GLI1*, *MEN1*, *MUTYH*, *PIK3CA*, *PTCH1*, *PTEN*, *SDHD*, *SHH*, *SMARCE1*, *SMO*, *SUFU*, *TAB3*, *TERT*, *TP53*, *TRAF7*, *TSC1*, *TSC2*, and *WRN*. These genes are reported as pertinent in schwannomas and other related tumors [5, 6].

Sequencing data was generated using the Illumina HiSeq 2000 sequencer and the 100-base pair paired-end sequencing protocol across rapid flow cell lanes. An analysis of the number of reads of each amplicon in the HaloplexHS data was performed with a mean coverage of 119 × with barcodes for the tumor sample and 278 × with barcodes for the blood sample. Somatic variants were initially called following GATK best practice using Mutect2, and false positives were filtered out by GATK FilterMutectCalls. On the other hand, germline variants were initially called following GATK best practice using Haplotypecaller, and potential false positives were filtered out by FilterVariantTranches after CNNScoreVariants with default parameters. Funcotator including ClinVar database was applied to annotate the remained variants. Sequencing reads were aligned to the human genome (hg19). Detailed conditions will be provided upon a reasonable request.

### Microsatellite analysis

Microsatellite analysis was also performed to detect 22q loss. Five microsatellite polymorphic markers flanking *NF2* were selected from the Genome Database and included D22S268, D22S1163, D22S929, D22S280, and D22S282. Detailed conditions of this analysis will be provided upon a reasonable request.

## Results

**Fig. 3:**
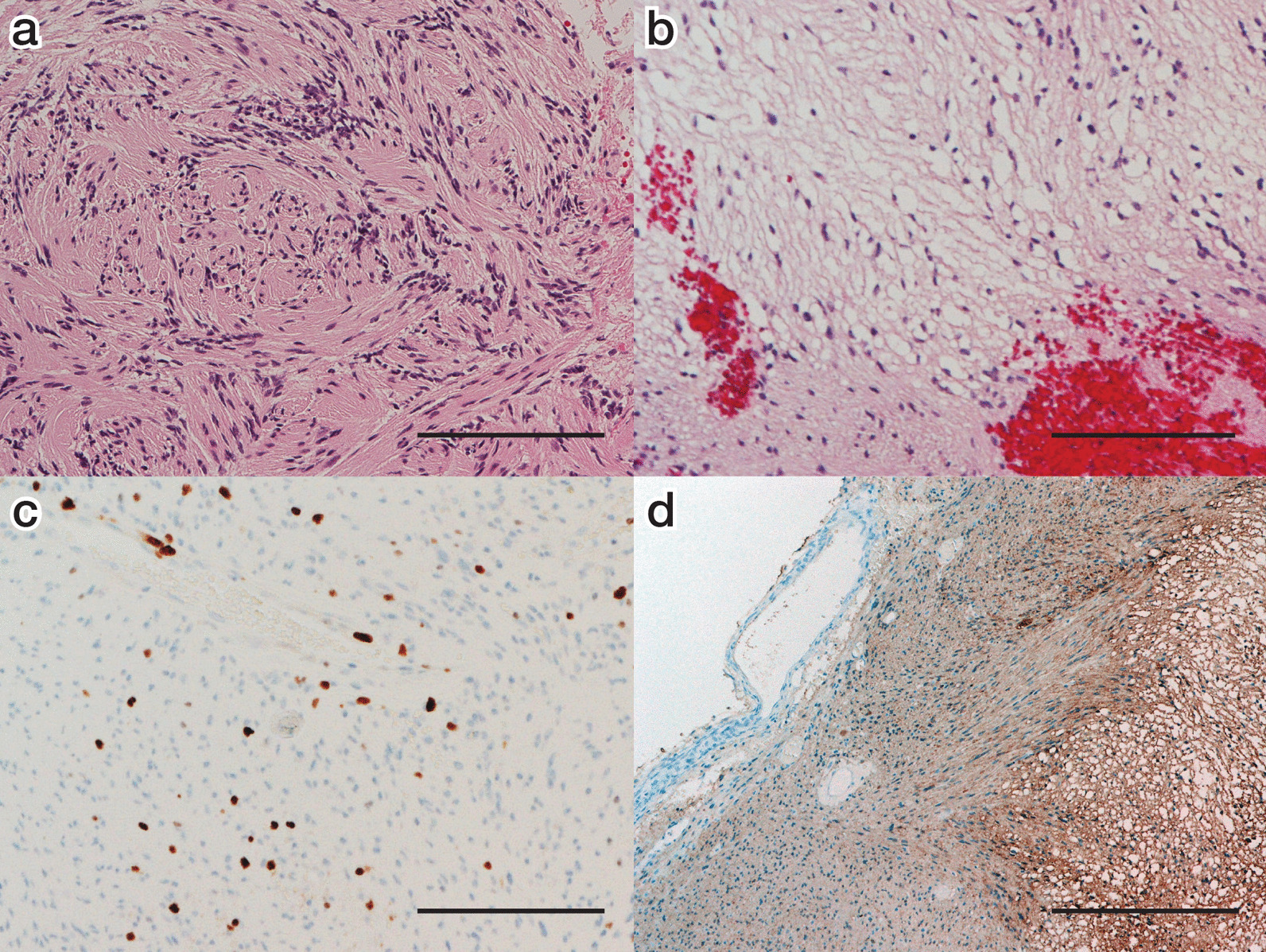
Photomicrographs of pathological specimens: **a** Compact spindle cells with rod-shaped nuclei and dense pericellular reticulin were aligned (Antoni A regions; hematoxylin and eosin; original magnification × 200; scale bar = 200 µm). We observed whorl formation in this area. **b** Stellate and spindle cells with smaller and hyperchromatic nuclei and scanty surrounding reticulin arranged in a myxoid stroma (Antoni B regions; hematoxylin and eosin; original magnification × 200; scale bar = 100 µm). **c** Tumor cells were stained with Ki-67 (original magnification × 200; scale bar = 100 µm). This area shows mitotic features with a Ki-67 index of 10–20%. **d** Tumor cells were diffusely positive for S100 (original magnification × 200; scale bar = 200 µm)

The final pathology report suggested brainstem intraparenchymal schwannoma. The specimen had typical characteristics of a schwannoma, such as Antoni A and B regions (Fig. 3a, b). The Ki-67 labeling index was 5%, but some components had a Ki-67 labeling index of 10–20% (Fig. 3c). Immunohistochemical staining for S100 protein was diffusely and intensely positive (Fig. 3d), whereas GFAP and EMA were negative in the tumor cells.

**Fig. 4:**
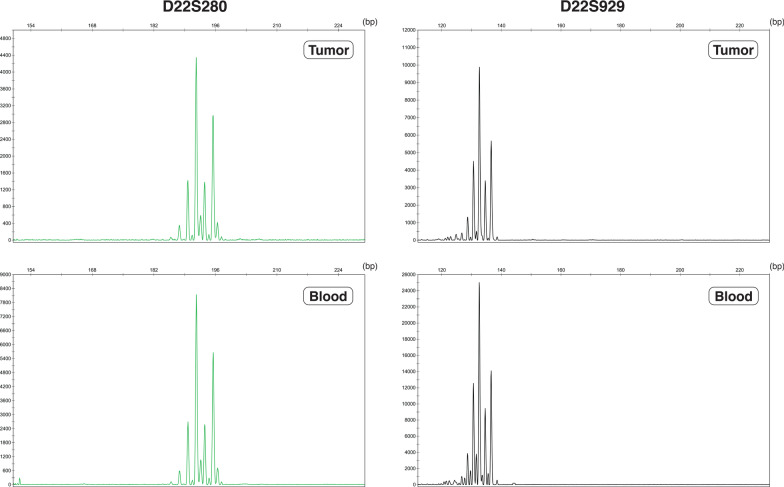
Two representative results of the microsatellite analysis. A comparison of tumor DNA and blood DNA using the flanking, D22S280, and intragenic, D22S929, microsatellite markers in the *NF2* gene region. No loss of heterozygosity by allele loss in the tumor DNA was detected

With regard to somatic variants, 29 variants were initially called, but 27 variants were filtered out as false positives by GATK FilterMutectCalls, and the remaining two indels were within the introns or the intragenic regions. As for germline mutations, 406 variants were initially called, and 10 of those were filtered out by FilterVariantTranches after CNNScoreVariants with default parameters. Of the remaining 396 variants, 69 were indels within the introns or the untranslated regions. None of the remining 327 single-nucleotide variants were pathogenic, according to the analysis by Funcotator including ClinVar database. In summary, no pathogenic variants were detected in *NF2*, *SMARCB1*, *LZTR1*, or the other targeted genes. The 22q loss was also not detected (Fig. 4). Regarding somatic copy number variants and exon deletions, reliable results could not be obtained.

## Discussion

We experienced the first case of brainstem schwannoma recurrence after resection, which was successfully controlled by irradiation. Intraparenchymal schwannomas are extremely rare, and few have been reported in the brainstem region. Some researchers have reported rapid regrowth after tumor resection [7–9], but there are no reports of postoperative relapse of brainstem schwannoma.

Only 14 case reports of brainstem intraparenchymal schwannoma have been published (Table 1) [2–4, 10–19]. According to a literature review by Gao et al. [18] of the 84 cases of intraparenchymal schwannoma, 23 cases occurred in the posterior fossa, and 9 in the brainstem. Interestingly, in almost all cases, the solid component was located anteriorly, and the cystic component was situated posteriorly. Furthermore, there was a bimodal patient distribution of adolescents and middle-aged individuals. Despite difficulty in the surgical approach and multiple adjacent critical structures, prognosis for patients with brainstem intraparenchymal schwannoma is relatively favorable. Moreover, prognosis for patients with overall intraparenchymal schwannoma is generally favorable [18]. However, three previous reports demonstrated rapid relapses [7–9]. The Ki-67 index is not mentioned in these reports. In our case, the Ki-67 index was 10–20% in the mitotic area. Given this pathological report, past malignant cases may have had similar findings. That is, we need to stay vigilant to detect postoperative recurrence if the pathology report indicates high Ki-67 index.

**Table 1:** Data of 14 cases obtained from previous reports plus our present case

Authors	Age (years)	Sex	Location	Extent of resection	Recurrence	Permanent neurological deficit	Other remarkable features
Prakash et al. [13]	14	F	Pons	Subtotal	–	Permanent facial palsy	
Aryanpur and Long [1]	50	F	Medulla–cervical cord	Gross total	–	No permanent deficit	
Ladouceur et al. [6]	46	F	Pons	Subtotal	–	Left-sided auditory impairment and left-sided hemiparesis	
Sharma and Newton [19]	18	M	Medulla	Subtotal	No description	Slight motor dysfunction (no details)	Surgery after empiric RT
Sharma et al. [18]	14	M	Medulla–cervical cord	Subtotal	Lost to F/U	Not described	
	14	M	Pons	Gross total	–	Good functional recovery (no details)	
Tanabe et al. [21]	68	F	Pons	Gross total	No description	Mild dysesthesia in the right hand	
Lee et al. [7]	29	F	Medulla–cervical cord	Gross total	Not available	(Died of aspiration pneumonia)	
Lin et al. [8]	48	M	Medulla	Gross total	–	Persistent ataxia	Surgery after empiric SRS
Muzzafar et al. [11]	68	M	Midbrain–pons	Subtotal	–	Trace left-sided trochlear palsy	
Srivastav et al. [20]	13	M	Pons	Subtotal	–	Able to perform routine activity with minimal support	Multiple neurofibromas in his father
Ramos et al. [14]	17	F	Midbrain–pons	Gross total	–	No permanent deficit	
Sharma et al. [17]	26	F	Medulla–cervical cord	Subtotal	–	Left-sided abducent and facial palsies	
Gao et al. [5]	12	F	Medulla	Gross total	–	No permanent deficit	Ki-67 labeling index: 1%
Present case report	21	M	Pons	Subtotal	+	Left-sided abducent and facial palsies	No tumor regrowth after RT

*F* female, *F/U* follow-up, *M* male, *RT* radiotherapy, *SRS* stereotactic radiosurgery

Some researchers speculate that specific soft membrane cells have the potential to transform into Schwann cells, which are the origin of intracerebral schwannomas [20]. Although the development of intraparenchymal schwannomas remains unclear, many researchers believe that intraparenchymal schwannomas originate from the subarachnoid space and peripheral venous plexus [21]. In fact, most intraparenchymal schwannomas are located in the superficial zone, such as on the cortical surface or in the paraventricular space. Neural crest cells directly differentiate into Schwann cell precursors that further transform into immature Schwann cells, which, at the stage of fetal birth, differentiate into demyelinated Schwann cells [22–26]. Hence, neural crest cells might be trapped in the brain parenchyma during embryonic development can give rise to intraparenchymal schwannomas.

Our genetic analysis of the tumor did not show any genetic alterations pertinent to schwannomas including *NF2* mutation or 22q loss of heterozygosity, whereas the genetic alteration of *NF2* gene occurs in 50 to 85% of sporadic vestibular schwannomas [5, 27–31]. To our knowledge, we described the result of genetic examination performed on brainstem schwannoma for the first time. Brainstem schwannomas may differ from conventional schwannomas (e.g., vestibular and spinal schwannoma) in terms of their genetic background, though further similar studies are essential to confirm this hypothesis.

We could not obtain reliable results on exonic deletions in the targeted genes and did not perform further investigation for the exonic deletions (e.g., multiplex ligation-dependent probe amplification). To clarify the genetic background of brainstem schwannomas, genome-wide comprehensive genetic analysis is essential. In addition, epigenetic analysis could also be important. Multiple studies on vestibular schwannomas showed that hypermethylation of the *NF2* locus, which leads to decreased expression of *NF2*, is a rare phenomenon [32, 33]. However, it does not necessarily mean *NF2* promoter methylation rarely occurs in brainstem schwannomas. Furthermore, in a previous report, genome-wide methylation profiling of vestibular and spinal schwannoma revealed distinct molecular subgroups of schwannoma, which were associated with the same anatomical locations [5]. Epigenetic factors may be involved in the pathogenesis of brainstem schwannomas, and further research on epigenetics is required.

## Conclusions

We experienced the first case of brainstem schwannoma recurrence after resection, which was successfully controlled by irradiation. To the best of our knowledge, this study is the first to report the results of a genetic examination of brainstem intraparenchymal schwannoma. Our genetic analysis revealed that the tumor did not have any somatic variants pertinent to schwannoma, such as in *NF2* and 22q loss. This case study suggests that brainstem schwannomas may differ from conventional schwannomas in their genetic background.

## Data Availability

The datasets used or analyzed during the current study are available from the corresponding author on reasonable request.

## Abbreviations

EMA: Epithelial membrane antigen
GFAP: Glial fibrillary acidic protein
MRI: Magnetic resonance imaging
POD: Postoperative day
